# 1% Isoflurane and 1.2 μg/ml of Propofol: A Combination of Anesthetics That Causes the Least Damage to Hypoxic Neurons

**DOI:** 10.3389/fnagi.2020.591938

**Published:** 2020-11-16

**Authors:** Xinyue Bu, Tang Li, Di Guo, Chenyi Yang, Jinxin Wang, Xinyi Wang, Zhuo Yang, Haiyun Wang

**Affiliations:** ^1^Department of Anesthesiology, The Third Central Clinical College of Tianjin Medical University, Tianjin, China; ^2^Tianjin Key Laboratory of Extracorporeal Life Support for Critical Diseases, Artificial Cell Engineering Technology Research Center, Tianjin Institute of Hepatobiliary Disease, Tianjin, China; ^3^Department of Anesthesiology, The Third Central Hospital of Tianjin, Tianjin, China; ^4^College of Medicine, State Key Laboratory of Medicinal Chemical Biology, Key Laboratory of Bioactive Materials for Ministry of Education, Nankai University, Tianjin, China; ^5^Department of Anesthesiology, The Third Central Clinical College of Tianjin Medical University, Tianjin, China

**Keywords:** binding immunoglobulin protein, endoplasmic reticulum stress, GABA_A_R α1 subunit, isoflurane, propofol

## Abstract

**Backgrounds**: Aging-related impairment of cerebral blood flow regulation leads to the disruption of neuronal micro-environmental homeostasis. Anesthetics should be carefully selected for aging patients since they have less cognition capacity. Effects and mechanisms of propofol or isoflurane have been widely investigated. However, how different combinations of propofol and isoflurane affect neurons and the mechanism still needs to be demonstrated.

**Methods**: We cultured rat hippocampal neurons and established a hypoxic injury model to imitate the micro-environment of aging brains. Three different combinations of propofol and isoflurane were applied to find out an optimum group *via* Cell Counting Kit-8 (CCK8) assay, lactic acid dehydrogenase (LDH) assay, real-time qPCR, and immunofluorescence of key proteins. Then BiP was silenced by small interfering RNA (siRNA) to explore the mechanism of how isoflurane and propofol affect neurons. Endoplasmic reticulum (ER) stress was measured by Western blot and immunofluorescence. To detect GABA_A_R α1 subunit proteostasis and its function, real-time qPCR, immunoprecipitation, and Western blot were carried out.

**Results**: Hypoxic neurons showed no different changes on cell viability, LDH leakage, and ER stress after treatment with 1% isoflurane and 1.2 μg/ml of propofol. Hypoxic neurons showed a sharp increase of LDH leakage and ER stress and a decrease of cell viability after treatment with 1.4% isoflurane and 0.6 μg/ml of propofol or 0.5% isoflurane and 1.8 μg/ml of propofol. After knockdown of BiP, the application of 1% isoflurane and 1.2 μg/ml of propofol led to the decrease of GABA_A_R α1 subunit protein content and viability of cell, as well as aggravation of ER stress.

**Conclusion**: A combination of 1% isoflurane and 1.2 μg/ml of propofol causes the least damage than do other dosages of both two drugs, and endogenous BiP plays an important role in this process.

## Introduction

Globally, 50% of all the elderly people are evaluated to undergo at least one surgical procedure. Evidence proves that about 25% of all the elderly having major surgery will have an identifiable fall in cognitive function, and 50% of these patients will suffer permanent damage (Dodds et al., 2017). Perioperative neurocognitive disorder (PND) is the overarching term for cognitive impairment in the preoperative or postoperative period and is associated with increased mortality (Evered et al., 2018). It is characterized as a decline in cognitive functions including memory, attention, information processing, and cognitive flexibility (Hovens et al., 2012). Aging has been reported as one of the major risk factors (Shoair et al., 2015). Aging elicits multifaceted functional impairment in cerebral microcirculation, leading to cerebral hypoperfusion, deprivation of brain oxygen and nutrition supply, oxidative injury, and neurovascular uncoupling (Daulatzai, 2017; Toth et al., 2017). Elder people have less reserve of neurological function and are less able to resist surgery- and anesthesia-induced cognitive impairment than younger people. As a result, when treating aging patients, special caution is needed to prevent surgery- and anesthesia-induced cognitive impairment with regard to the choice and depth of anesthesia, dosage, and duration of perioperative anesthetics, and surgical strategy.

Propofol (2,6-diisopropylphenol) is a widely used intravenous anesthetic agent (Zhong et al., 2018). In addition to its sedative effects, propofol has a protective effect against cerebral ischemia–reperfusion injury in animal models, which can reduce infarction size and improve neurologic scores (Li et al., 2014; Shi et al., 2014). More specifically, propofol at a subanesthetic dose has a neuroprotective effect on cerebral ischemia–reperfusion rats, but not at higher doses (Wang et al., 2009). Isoflurane is an isomeride of enflurane, which is commonly used in inhalation anesthesia, but it can also induce neurogenetic damage and neurocognitive disorder and even accelerate the process of Alzheimer’s disease (Perucho et al., 2010; Zuo et al., 2018). Neurotoxicity of isoflurane is positively correlated with dose and duration (Wei et al., 2008; Wang H. et al., 2014). Previous studies showed that isoflurane minimum alveolar concentration (MAC) value was 1.45 ± 0.17%; 1.9% isoflurane, equivalent to 1.3 MAC, was sufficient to induce general anesthesia in rats (Boruta et al., 2012), while a minimal infusion rate at 40 mg·kg^−1^·h^−1^ was required using propofol alone to induce general anesthesia in rats (Logginidou et al., 2003). Our previous study confirmed that a single use of anesthetic dose propofol (40 mg·kg^−1^·h^−1^) or isoflurane (1.9%) aggravated cognitive impairment of aging rats with cerebral hypoperfusion, while a combination of sub-anesthesia dose propofol and isoflurane (1% isoflurane plus 20 mg·kg^−1^·h^−1^ propofol) did not (Bu et al., 2020). However, the effects of different combinations of propofol and isoflurane on hippocampal neurons remain to be explored.

The endoplasmic reticulum (ER) is a vast membranous network and a unique environment for protein folding, secretion, lipid biosynthesis, and calcium homeostasis (Kim et al., 2008), which are all required for maintaining normal cell function. ER stress is a subcellular pathological process of impairment in ER homeostasis. A number of insults have been shown to induce protein misfolding in the ER and cause ER stress, such as ischemia, nutrient deprivation, alterations in calcium concentrations, and oxidative stress (Martindale et al., 2006; Minamino et al., 2010; Wang et al., 2011). ER stress has been associated with isoflurane-induced cognitive impairment and neurodegenerative conditions such as Alzheimer’s disease (Cai et al., 2014; Ge et al., 2015; Xiang et al., 2017). A previous study also revealed that ER stress is involved in the neuroprotection of propofol (Wang L. et al., 2014). It remains unknown whether ER stress is involved in the effect of combined use of isoflurane and propofol on neurons. Furthermore, ER-localized molecular chaperone, BiP, one of the heat shock protein-70 family, has protective effects by attenuating ER stress (Feaver et al., 2008; Fu et al., 2008). BiP can be induced by oxidative stress (Dickhout et al., 2005). Therefore, we also investigated the involvement of BiP in the combined effects of isoflurane and propofol.

Synaptic inhibition in the brain mainly relies on GABA signaling. The GABA_A_ receptors (GABA_A_Rs) are the major inhibitory receptors in the central nervous system and can mediate fast postsynaptic inhibitory effects. The α1 subunit-typed GABA_A_R is the most abundant composition subtype (Liu and Wong-Riley, 2004), and the α1 subunit is the key to GABA_A_R activity (Williams and Akabas, 2002; Kelley et al., 2008). GABA_A_Rs are assembled from their component subunits in the ER (Jacob et al., 2008); thus, protein homeostasis of GABA_A_R α1 subunit was used as an evaluation index for functional damage of neurons.

Therefore, the purpose of this study was to compare the effects of three different combination methods of propofol and isoflurane on hypoxic hippocampus neurons and to screen out the combination method with the least damage, so as to provide a safer general anesthesia strategy for patients with fragile cognitive function in clinical work; and the potential mechanism is discussed to provide a reference for subsequent basic scientific research.

## Materials and Methods

### Hippocampal Neuron Culture and Treatment

Primary hippocampal neuronal cultures from 16- to 18-day-old Sprague–Dawley rat embryos were prepared as described previously (Kaech and Banker, 2006), with modifications. Briefly, the hippocampus was removed and dissociated into a single-cell suspension. Neurons were plated at an average density of 5 × 10^5^ cells/ml in supplemented Neurobasal medium on poly-D-lysine-coated glass coverslips. Neurons were maintained at 37°C in a humidified atmosphere of 95% air/5% CO_2_ and were used 7 days later. The confluency of cells was 80–90%. The purity of the cultured primary hippocampal neurons was determined by immunocytochemistry with an antibody against microtubule-associated protein-2 (MAP-2). The percentage of cultured neurons was above 95 ± 2.6% (Supplementary Figure 1). For each group, the operations were replicated six times.

### Antibodies

The primary antibodies include anti-MAP-2 (1:1,000, Abcam, catalog no. ab32454) anti-GABA_A_R a1 (1:1,000, Abcam, catalog no. ab94585), anti-BiP (1:1,000, Abcam, catalog no. ab21685), anti-CHOP (1:500 for immunofluorescence, 1:1,000 for Western blotting, Cell Signaling Technology, catalog no. 2895), anti-β-actin (1:1,000, Abcam, catalog no. ab8226), anti-pan-cadherin (1:1,000, Abcam, catalog no. ab16505), anti-β-tubulin (1:1,000, Abcam, catalog no. ab18207), and anti-ubiquitin (1:1,000, Abcam, catalog no. ab134953). The secondary antibodies include Goat Anti-Mouse IgG H&L [horseradish peroxidase (HRP)] (1:2,000, Abcam, catalog no. ab205718), Goat Anti-Mouse IgG H&L (HRP; 1:2,000, Abcam, catalog no. ab250719), Goat Anti-Rabbit IgG H&L (Alexa Fluor^®^ 488; 1:1,000 for immunofluorescence, ab150077), and Goat Anti-Mouse IgG H&L (Alexa Fluor^®^ 594; 1:1,000 for immunofluorescence, ab150120).

### Grouping 1

To investigate the effects of different dosage for isoflurane and propofol on the cell viability and cytotoxicity, hippocampal neurons were cultured in five groups (*n* = 6/group): (1) control group (C); (2) hypoxia-injured group (H); (3) hypoxia-injured cells treated with 1% isoflurane and 1.2 μg/ml propofol group (IP_1_); (4) hypoxia-injured cells treated with 1.4% isoflurane and 0.6 μg/ml propofol group (IP_2_); and (5) hypoxia-injured cells treated with 0.5% isoflurane and 1.8 μg/ml propofol group (IP_3_).

### Grouping 2

To explore the role of BiP on the hippocampal neurons treated by the combination of 1% isoflurane and 1.2 μg/ml of propofol, small interfering RNA (siRNA) was performed at 3 days* in vitro* (DIV) for 72 h. The transfected hippocampal neurons were cultured in six groups (*n* = 6 for each group): (1) control-transfected cells (Con-siRNA + C) group; (2) hypoxia-treated control-transfected cells (Con-siRNA + H) group; (3) hypoxia-treated control-transfected cells treated with 1% isoflurane and 1.2 μg/ml of propofol (Con-siRNA + IP_1_) group; (4) control BiP-transfected cells (BiP-siRNA + C) group; (5) hypoxia-treated BiP-transfected cells (BiP-siRNA + H) group; and (6) hypoxia-treated BiP-transfected cells treated with 1% isoflurane and 1.2 μg/ml of propofol (BiP-siRNA + IP_1_) group.

### Anoxic Treatment and Anesthesia

To mimic the ischemia–hypoxia condition, the neurons in the Con-siRNA + H, Con-siRNA + IP_1_, BiP-siRNA + H, and BiP-siRNA + IP_1_ groups were subjected to hypoxia (Hofmeijer et al., 2014). Briefly, at 7 DIV, primary hippocampal neurons were placed in an incubator at 37°C with 5% CO_2_, 3% O_2_, and 92% N_2_ for 3 h. After hypoxia treatment, 1.2 μg/ml of propofol was added to the culture medium in the Con-siRNA + IP_1_ and BiP-siRNA + IP_1_ groups. Then, the culture plates were immediately placed in an airtight and thermostatic chamber with internal electric fans and inlet and outlet valves (Benzonana et al., 2013). Isoflurane was delivered to the chamber at a rate of 2 l/min using a vaporizer with 5% CO_2_, 21% O_2_, and 74% N_2_. An anesthetic analyzer (Datex-Ohmeda, UK) was used to monitor the effluent isoflurane concentration. The concentration of isoflurane was maintained at 1% for 3 h. The cells were then exposed to the fresh neuronal culture medium and normoxic conditions (95% air, 5% CO_2_) for 24 h. In other groups, the hippocampal neurons were placed in the incubator under the condition of 5% CO_2_, 21% O_2_, and 74% N_2_ balanced.

### RNA Interference

SiRNA against rat BiP (targeting sense: 5′-GAGGCGUAUUUGGGAAAGATT-3′; antisense: 5′-UCUUUCCCAAAUACGCCUCTT-3′) and a control siRNA (targeting sense: 5′-UUCUCCGAACGUGUCACGUTT-3′; antisense: 5′-ACGUGA CACGUUCGGAGAATT-3′) were purchased from GeneChem Company (Shanghai, China). The siRNAs were transfected into neurons using lentivirus at 3 DIV (Wong and Lazinski, 2002). After 8 h, the medium was replaced, and the cells were incubated at 37°C for 72 h. Western blot was used to confirm the BiP silencing.

### Cell Viability Assay

Cell viability was assessed by using the Cell Counting Kit-8 (CCK8; Dojindo Laboratories, Kumamoto, Japan) according to the manufacturer’s instructions. Briefly, cells were cultured in 96-well plates; 24 h after the anesthetic treatment, 10 μl of CCK8 solution was added to each well, followed by 1 h of incubation at 37°C. The absorbance values were measured at 450 nm by using a microplate reader (Elx 800, Bio-TEK, Winooski, VT, USA).

### Lactic Acid Dehydrogenase Release

Twenty-four hours after the anesthetic treatment, lactic acid dehydrogenase (LDH) release was detected by using the LDH Assay kit (Abcam, UK). The cell culture plates were centrifuged at 600 *g* for 10 min, and supernatants (10 μl/well) were extracted into another 96-well-plate. Then, a 100  μl LDH reaction mix was added to each well and incubated at room temperature for 30 min. The absorbance values were measured at 490 nm on the microplate reader (BioTek, Winooski, VT, USA).

### Quantitative Real-Time Polymerase Chain Reaction

The expression of mRNA was detected by qRT-PCR. Total RNA was extracted from cells according to the protocols of the manufacturers and purified with RNA 4 Aqueous kit (Ambion Inc., Austin, TX, USA). Total RNA concentration was measured by spectrophotometer (Thermo Fisher Scientific, Waltham, MA, USA). cDNA was synthesized using a PrimeScript RT reagent kit (Takara, Japan). Then, cDNA was used as a template for qPCR with Premix Ex TaqII (Takara, Japan) on Applied Biosystems 7500 RT-PCR System (Applied Biosystems, Foster City, CA, USA). The mRNA levels were normalized to GAPDH. Relative quantification was achieved by the comparative 2^ΔΔ^ method (Schmittgen and Livak, 2008). The nucleotide sequences of the PCR primers (Sangon Biotech, Shanghai, China) are as follows:

- XBP1s mRNA: (forward 5′-GATGAATGCCCTGGTTACTG-3′;
- reverse 5′-AGATGTTCTGGG GAGGTGAC-3′)
- ATF6 mRNA: (forward 5′-AAGTGAAGAACCATTACTTTATATC-3′;
- reverse 5′-TTTCTGCTGGCTATTTGT-3′)
- ATF4 mRNA: (forward 5′-CATTCCTCGATTCCAGCAAAGCAC-3′;
- reverse5′-TTCTCCAACATCCAATCTGTCCCG-3′)
- GABA_A_R α1 mRNA: (forward 5′-TGTCTTTGGAGTGACGACC-3′;
- reverse 5′-ATCCCACGCATACCCTCTCT-3′)
- GRPDH mRNA: (forward 5′-AACAGCAACTCCCACTCTTC-3′;
- reverse 5′-CCTCTCTTGCTCAGTGTCCT-3′)

### Immunofluorescence

Primary antibodies used were anti-MAP-2 and anti-CHOP. Staining was visualized with a Zeiss LSM 510 Meta confocal system (10×, 20×, and 40× objectives), and a 405-nm diode laser, a 488-nm Ar laser, and a 594-nm HeNe laser were used for excitation of fluorophores.

### Co-immunoprecipitation

Cell proteins were extracted by Pierce™ Classic Magnetic IP/Co-IP Kit (Thermo Fisher Scientific, Waltham, MA, USA, 88804) according to the manuals. Protein co-immunoprecipitation (Co-IP) was performed by using Pierce™ Classic Magnetic IP/Co-IP Kit as well. Protein samples were run on 8% sodium dodecyl sulfate–polyacrylamide gel electrophoresis (SDS–PAGE) gel and probed with appropriate antibodies by Western blotting.

### Western Blot

To obtain total cellular protein, neurons were lysed at 4°C in radioimmunoprecipitation assay (RIPA) buffer (Solarbio, R0010) mixed with phenylmethylsulfonyl fluoride (PMSF; Solarbio, P0100). Membrane protein fractions were obtained with a Mem-PER Plus Membrane Protein Extraction Kit (Thermo Fisher Scientific, Waltham, MA, USA). Protein lysates were resolved by SDS-PAGE gel and transferred to Immun-Blot polyvinylidene difluoride (PVDF) membranes (Solarbio). Membranes were incubated in blocking buffer (5% milk, 0.1% Tween20 in Tris-buffered saline) for 1 h and probed overnight with primary antibody at 4°C. Blots were rinsed thrice (0.1% Tween20 in Tris-buffered saline, 5 min each), followed by incubation with peroxidase-conjugated secondary antibody for 2 h at room temperature. Bands were visualized by exposing blots to X-ray film after incubation with freshly made chemiluminescent reagent (EMD Millipore, Billerica, MA, USA) and were quantified using Image Pro Plus.

### Statistical Analysis

The data were analyzed with SPSS 24 (SPSS Science Inc.; Chicago, IL, USA). Data are presented as mean ± standard deviation (SD). All data were analyzed using one-way ANOVA with Tukey *post hoc* comparisons. *P* < 0.05 was the criterion for statistical significance.

## Results

### 1% Isoflurane and 1.2 μg/ml of Propofol Caused the Least Damage to the Hypoxic Neurons

**Figure 1:**
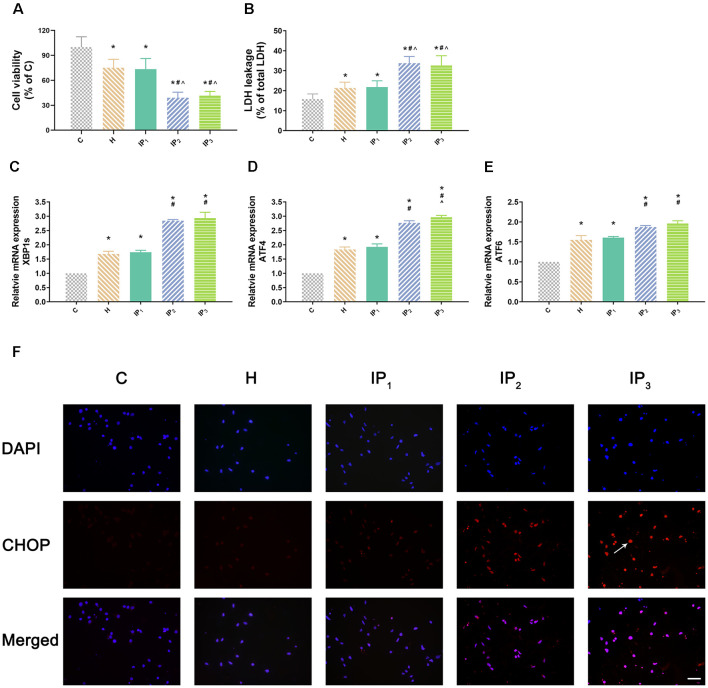
One percent isoflurane and 1.2 μg/ml of propofol caused the least damage to the hypoxic neurons.** (A)** Statistical graph of the neurons viability determined by the Cell Counting Kit-8 (CCK-8) assay. Data are expressed as the mean ± standard deviation (SD; *n* = 6/group). Note that hypoxia resulted in a significant decrease in cells viability. IP_2_ and IP_3_ aggravated this injury but IP_1_ did not. **(B)** Statistical graph of the neurons cytotoxicity determined by the lactic acid dehydrogenase (LDH) assay. Data are expressed as the mean ± SD (*n* = 6/group). Note that hypoxia resulted in a significant increase in cells cytotoxicity. IP_2_ and IP_3_ aggravated this injury but IP_1_ did not. **(C–E)** mRNA level analysis of XBP1s, ATF4, and ATF6 in neurons. Data are expressed as the mean ± SD (*n* = 6/group). Note that hypoxia resulted in a significant increase in transcription of XBP1s, ATF4, and ATF6. IP_2_ and IP_3_ aggravated this injury but IP_1_ did not.** (F)** Immunofluorescent microscopy for CHOP. Nuclear was stained by DAPI; CHOP antibody was marked by rhodamine Red-X. Note that IP_2_ and IP_3_ caused an increase in expression of CHOP but IP_1_ did not. *P < 0.05 compared with group C; ^#^*P* < 0.05 compared with group H; ^∧^*P* < 0.05 compared with group IP_1_. Scale bars = 200 μm.

First, hippocampal neurons were exposed to hypoxic condition to mimic aging-related cerebral hypoperfusion. Cell viability and cytotoxicity were evaluated to explore the effects of the different drug combinations. Compared with that of the normal neurons (group C: 100.00 ± 12.42), the cell viability of hypoxia-injured neurons decreased significantly (group H: 75.23 ± 10.04, *P* < 0.05). Compared with the cell viability of group H, no significant change of cell viability was found in group IP_1_ (hypoxia-injured cells treated with 1% isoflurane and 1.2 μg/ml propofol group; group H vs. IP_1_: 75.23 ± 10.04 vs. 73.48 ± 12.73, *P* > 0.05). Cell viability decreased distinctly in group IP_2_ (hypoxia-injured cells treated with 1.4% isoflurane and 0.6 μg/ml propofol group; 39.02 ± 6.75, *P* < 0.05) and IP_3_ (hypoxia-injured cells treated with 0.5% isoflurane and 1.8 μg/ml propofol group; 41.50 ± 5.21, *P* < 0.05; Figure 1A) compared with group H. Meanwhile, anoxia resulted in ascension of cytotoxicity in hippocampal neurons (C vs. H: 15.85 ± 2.54 vs. 21.35 ± 2.93, *P* < 0.05). There was no significant difference in cytotoxicity between group H and IP_1_ (H vs. IP_1_: 21.35 ± 2.93 vs. 21.79 ± 3.19, *P* > 0.05). However, in comparison with group H, cytotoxicity increased sharply in group IP_2_ (39.02 ± 6.75, *P* < 0.05) and IP_3_ (32.66 ± 4.85, *P* < 0.05; Figure 1B).

### 1% Isoflurane and 1.2 μg/ml of Propofol Induced the Least Endoplasmic Reticulum Stress

**Figure 2:**
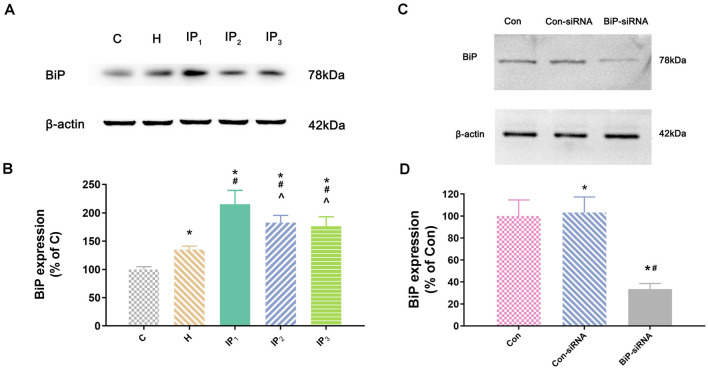
The expression of BiP in hippocampal neurons.** (A)** The expression of BiP in hippocampal neurons was determined by Western blotting. **(B)** Statistical graph of the expression of BiP. Note that treatment with isoflurane and propofol caused distinctly increase in BiP expression of hypoxic neurons. Data are expressed as the mean ± SD (*n* = 6/group). **(C)** Verifying siRNA targeting BiP on BiP protein expression. BiP-siRNA significantly down-regulates BiP protein expression. **(D)** Statistical graph of the expression of BiP. Data are expressed as the mean ± SD (*n* = 6/group). *P < 0.05 compared with group Con; ^#^*P* < 0.05 compared with group Con-siRNA; ^∧^*P* < 0.05 compared with group IP_1_.

Second, we detected the expression of the element of the ER stress canonical biomarkers: XBP1, ATF4, and ATF6. Results showed that hypoxia increased the mRNA expression of XBP1, ATF4, and ATF6. Compared with that in the control group, mRNA expression of XBP1 (1.68 ± 0.09, *P* < 0.05), ATF4 (1.83 ± 0.09, *P* < 0.05), and ATF6 (1.55 ± 0.11, *P* < 0.05) increased in group H. Compared with that in group H, mRNA expression of XBP1 did not change significantly in group IP_1_ (1.74 ± 0.07, *P* > 0.05) but increased distinctly in groups IP_2_ (2.84 ± 0.04, *P* < 0.05) and IP_3_ (2.94 ± 0.20, *P* < 0.05; Figure 1C). The expression of ATF4 mRNA did not change significantly in group IP_1_ (1.92 ± 0.10, *P* > 0.05) but increased distinctly in groups IP_2_ (2.76 ± 0.08, *P* < 0.05) and IP_3_ (2.97 ± 0.06, *P* < 0.05; Figure 1D). Likewise, the expression of ATF6 mRNA did not change significantly in group IP_1_ (1.61 ± 0.03, *P* > 0.05) but increased distinctly in groups IP_2_ (1.87 ± 0.04, *P* < 0.05) and IP_3_ (1.96 ± 0.07, *P* < 0.05; Figure 1E). CHOP, a pro-apoptotic transcription factor, plays a critical role in ER stress-induced apoptosis (Biwer and Isakson, 2017). Results of immunofluorescence showed that expression of CHOP rose visibly in groups IP_2_ and IP_3_ (Figure 1F). The expression of BiP protein increased distinctly in group H (134.94 ± 6.09, *P* < 0.05) compared with group C (100.00 ± 4.79). Compared with that in group H, the expression of BiP protein increased distinctly in groups IP_1_ (215.39 ± 24.17, *P* < 0.05), IP_2_ (182.78 ± 12.66, *P* < 0.05), and IP_3_ (176.36 ± 16.78, *P* < 0.05; Figures 2A,B). BiP is an indicator that can relieve ER stress. Compared with that in group H, the expression of BiP in group IP_2_ and IP_3_ also increased, but it was far less than that in group IP_1_. In combination with other indicators of ER stress, we concluded that the combination of 1% isoflurane and 1.2 μg/ml of propofol induced ER stress was the least among the three compound drug groups.

### Damage to the Hypoxic Neurons Caused by Anesthetics Was Alleviated by Raising Endogenous Binding Immunoglobulin Protein

To identify the mechanisms by which 1% isoflurane and 1.2 μg/ml of propofol caused the least damage to the hypoxic neurons, we determined the effect of endogenous BiP by using of BiP-siRNA in rat hippocampal neurons. Western blot showed that in comparison with control group or lentiviral vector-negative control treated neurons, the expression of BiP protein was significantly dampened in the BiP-siRNA transfected cells (Con-siRNA vs. BiP-siRNA: 103.16 ± 14.20 vs. 33.53 ± 5.06, *P* < 0.05; Figures 2C,D).

**Figure 3:**
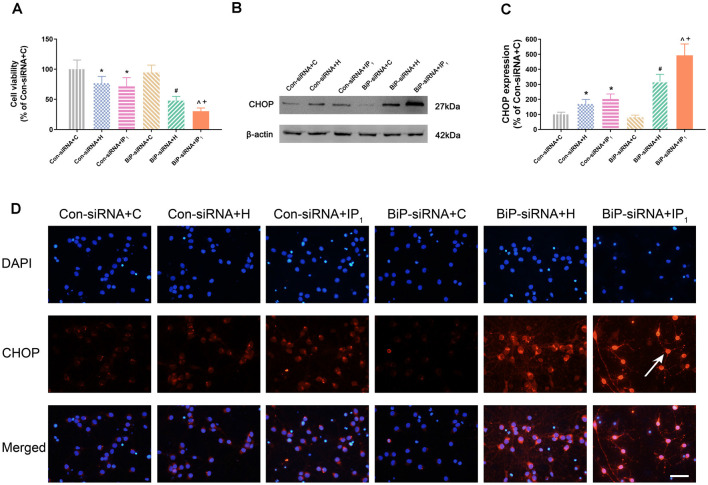
Knockdown of BiP by siRNA aggravated the neuronal injury and endoplasmic reticulum (ER) stress caused by anesthetics. **(A)** Statistical graph of the neurons viability determined by the Cell Counting Kit-8 (CCK8) assay. Data are expressed as the mean ± SD (*n* = 6/group). **(B,C)** The expression of CHOP in hippocampal neurons was determined by Western blotting. Data are expressed as the mean ± SD (*n* = 6/group). One percent isoflurane and 1.2 μg/ml of propofol significantly up-regulated CHOP protein expression in neurons after treatment with BiP siRNA. *P < 0.05 compared with group Con-siRNA + C; ^#^*P* < 0.05 compared with group Con-siRNA + H; ^∧^*P* < 0.05 compared with group Con-siRNA + IP_1_; ^+^*P* < 0.05 compared with group BiP-siRNA + H. **(D)** Immunofluorescent microscopy for CHOP. Nuclear was stained by DAPI; CHOP antibody was marked by rhodamine Red-X. Scale bars = 50 μm.

No significant change of the viability of neurons was found between Con-siRNA + H and Con-siRNA + IP_1_ groups (Con-siRNA + H vs. Con-siRNA + IP_1_: 76.80 ± 11.21 vs. 71.60 ± 14.96, *P* > 0.05; Figure 3A). However, the viability of neurons in the BiP-siRNA + IP_1_ group was significantly reduced in comparison with that of the BiP-siRNA + H group (BiP-siRNA + IP_1_ vs. BiP-siRNA + H: 30.35 ± 5.38 vs. 47.81 ± 7.17, *P* < 0.05; Figure 3A). BiP siRNA interference caused obvious damage of IP_1_ on hypoxic neurons, which suggested that 1% isoflurane and 1.2 μg/ml of propofol affected neurons by raising endogenous BiP.

### Endoplasmic Reticulum Stress-Related Apoptosis Caused by Anesthetics Was Alleviated by Raising Endogenous Binding Immunoglobulin Protein

There was no difference in the expression of CHOP between the Con-siRNA + H and Con-siRNA + IP_1_ groups (Con-siRNA + H vs. Con-siRNA + IP_1_: 168.97 ± 30.67 vs. 202.73 ± 33.55, *P* > 0.05; Figures 3B–D). However, compared with that in the BiP-siRNA + H group, the expression of CHOP raised significantly in the BiP-siRNA + IP_1_ group (BiP-siRNA + IP_1_ vs. BiP-siRNA + H: 492.91 ± 75.60 vs. 318.79 ± 47.82, *P* < 0.05; Figures 3B–D).

### Disturbance of γ-Aminobutyric Acid A Type Receptor α1 Subunit Proteostasis Caused by Anesthetics Was Alleviated by Raising Endogenous Binding Immunoglobulin Protein

**Figure 4:**
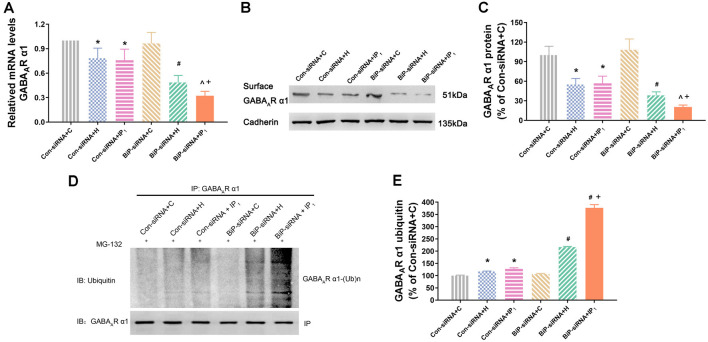
Knockdown of BiP by siRNA aggravated the disturbance of GABA_A_R α1 subunit protein homeostasis caused by anesthetics. **(A)** mRNA level analysis of GABA_A_R α1 subunit in neurons. Note that 1% isoflurane and 1.2 μg/ml of propofol resulted in a significant decrease of GABA_A_R α1 subunit transcription in neurons after treatment with BiP siRNA. **(B,C)** The expression of GABA_A_R α1 subunit in hippocampal neurons was determined by Western blotting. Note that 1% isoflurane and 1.2 μg/ml of propofol resulted in a significant decrease of GABA_A_R α1 subunit expression in neurons after treatment with BiP siRNA. **(D,E)** Ubiquitin GABA_A_R α1 of expression level increased in hippocampal neurons was determined by immunoprecipitation and Western blotting. Note that 1% isoflurane and 1.2 μg/ml of propofol resulted in a significant increase of GABA_A_R α1 subunit degradation in neurons after treatment with BiP siRNA. Data are expressed as the mean ± SD (*n* = 6/group). *P < 0.05 compared with group Con-siRNA + C; ^#^*P* < 0.05 compared with group Con-siRNA + H; ^∧^*P* < 0.05 compared with group Con-siRNA + IP_1_; ^+^*P* < 0.05 compared with group BiP-siRNA + H.

We tested the mRNA expression of GABA_A_R α1 in the hippocampal neurons by RT-qPCR assay. We found that there was no difference in the mRNA expression of GABA_A_R α1 between the Con-siRNA + H and Con-siRNA + IP_1_ groups (Con-siRNA + H vs. Con-siRNA + IP_1_: 78.29 ± 12.34 vs. 75.84 ± 13.51, *P* > 0.05; Figure 4A). However, compared with that in the BiP-siRNA + H group, the mRNA expression of GABA_A_R α1 was significantly down-regulated in the BiP-siRNA + IP_1_ group (BiP-siRNA + H vs. BiP-siRNA + IP_1_: 48.65 ± 8.49 vs. 32.27 ± 5.40, *P* < 0.05; Figure 4A). It suggests that transcription was inhibited when neurons were treated with 1% isoflurane and 1.2 μg/ml of propofol after BiP was knocked down.

Similarly, western blot analysis showed that no significant change of the expression of GABA_A_R α1 protein was found between Con-siRNA + H and Con-siRNA + IP_1_ groups (Con-siRNA + H vs. Con-siRNA + IP_1_: 54.93 ± 9.37 vs. 56.82 ± 10.91, *P* > 0.05; Figures 4B,C). However, compared with that in the BiP-siRNA + H group, the expression of GABA_A_R α1 protein reduced significantly in the BiP-siRNA + IP_1_ group (BiP-siRNA + H vs. BiP-siRNA + IP_1_: 38.31 ± 5.29 vs. 20.55 ± 2.93, *P* < 0.05; Figures 4B,C).

To determine whether combination of 1% isoflurane and 1.2 μg/ml of propofol influenced the proteasome degradation of the GABA_A_R α1 subunit, hippocampal neurons were immunoprecipitated using anti-α1 antibody and blotted for ubiquitin. We found no significant change of the intensity of ubiquitinated GABA_A_R α1 subunit between the Con-siRNA + H and Con-siRNA + IP_1_ groups (Con-siRNA + H vs. Con-siRNA + IP_1_: 116.71 ± 1.74 vs. 127.12 ± 4.77, *P* > 0.05; Figures 4D,E). However, compared with that in the BiP-siRNA + H groups, the intensity of ubiquitinated GABA_A_R α1 subunit increased distinctly in the BiP-siRNA + IP_1_ group (BiP-siRNA + H vs. BiP-siRNA + IP_1_: 216.36 ± 3.08 vs. 376.62 ± 13.13, *P* < 0.05; Figures 4D,E). The results revealed that 1% isoflurane and 1.2 μg/ml of propofol attenuated proteasome degradation of the GABA_A_R α1 subunit by increasing endogenous BiP.

## Discussion

Our data demonstrate that 1% isoflurane and 1.2 μg/ml of propofol have a neuroprotective effect, which is related to the up-regulation of an ER resident chaperone, BiP. BiP expression is thought to be a key cellular component of this effect, since the protective effect of 1% isoflurane and 1.2 μg/ml of propofol was abolished by knocking down endogenous BiP through siRNA.

Aging is a key factor that contributes to cerebral hypoperfusion (Toth et al., 2017), which is emerging as a major contributor to cognitive decline and degenerative processes leading to dementia (Alsop et al., 2010; Chao et al., 2010). Animal study has proved that chronic cerebral hypoperfusion caused by severe bilateral carotid stenosis led to mild cognitive impairment and slightly structural changes in the brains of aged rats (Wang et al., 2020). It has been generally accepted that the metabolic demand by local neuronal-glial activity for oxygen and glucose is tightly coupled to cerebral blood flow delivery (Roy and Sherrington, 1890). Thus, in this study, we cultured rat hippocampal neurons under hypoxia condition to imitate the microenvironment change of aging brains. After hypoxia, cell viability decreased and LDH leakage increased significantly, which could confirm that hypoxia induced neuron damage.

Previous studies showed that 1.9% isoflurane, equivalent to 1.3 MAC, was sufficient to induce general anesthesia in rats (Boruta et al., 2012), while a minimal infusion rate at 40 mg·kg^−1^·h^−1^ was required using propofol alone to induce general anesthesia in rats (Logginidou et al., 2003). The infusion rate of 20 mg·kg^−1^·h^−1^ led to an estimated mean propofol plasma concentration of 1.2 μg/ml. Therefore, in our study, doses were carefully selected combining isoflurane and propofol (1% and 1.2 μg/ml, 1.4% and 0.6 μg/ml, or 0.5% and 1.8 μg/ml) to imitate general anesthesia *in vivo*.

A large number of previous studies have demonstrated the dose-dependent effects of propofol and isoflurane. *In vitro* study showed that a high dose of isoflurane (treatment at a dose of 2% for 6 h) induced apoptosis by causing ER stress but a lower dose isoflurane (treatment at a dose of 1% for 1, 3, and 6 h) did not (Wang et al., 2014). *In vivo* study suggested that isoflurane (treatment at a dose of 1.3% for 4 h) caused cognitive impairment in aged rats. Inhibition of ER stress overactivation contributed to the relief of isoflurane-induced histopathologic changes (Ge et al., 2015). Moreover, Coghlan et al. (2018) confirmed that the effect of isoflurane was dose dependent, showing no statistical difference from control in aggregated, mislocalized protein at 0.5 MAC, an intermediate response at 0.75 MAC, and the most significant response at 1.0 MAC. Our previous study showed that propofol at doses of 10 or 20 mg·kg^−1^·h^−1^ infused at the onset of reperfusion for 30 min could provide neuroprotection to transient middle cerebral artery occlusion rats but 30 mg·kg^−1^·h^−1^ could not (Wang et al., 2009). Thal et al. (2014) showed that infusion of propofol (36 or 72 mg·kg^−1^·h^−1^) resulted in aggravation of neurologic dysfunction, increased 28-day mortality rate, and impaired posttraumatic neurogenesis. *In vitro* study demonstrated that the neuroprotective effect of propofol increased in a dose-dependent manner within 10 μM and decreased in a dose-dependent manner beyond 10 μM. The increase of endogenous BiP was the key to propofol’s neuroprotection (Wang L. et al., 2014). All this evidences could prove that a single high dose of propofol or isoflurane may cause neuron damage and cognitive impairment, which aroused our interest in studying low-dose combination applications. Our previous *in vivo* study revealed that combination of sub-anesthetic dose isoflurane and propofol (1% isoflurane plus 20 mg·kg^−1^·h^−1^ propofol) did not cause cognitive impairment of aged rats with cerebral hypoperfusion as compared with single-use of propofol (20 mg·kg^−1^·h^−1^) or isoflurane (1.9%; Bu et al., 2020). In the present study, we compared the effects of three different dosages of isoflurane and propofol (1% and 1.2 μg/ml, 1.4% and 0.6 μg/ml, or 0.5% and 1.8 μg/ml) on primary hypoxic hippocampal neurons with the aim of finding the way to minimize the damage caused by anesthetic to vulnerable neurons. In fact, no matter which combination is chosen, anesthetics are unlikely to reverse the damage to neurons caused by hypoxia. All we can do is find a way to administer drugs so that anoxic neurons are not further damaged by anesthetics. Compared with those in the hypoxia group (group H), the neuronal injury indexes of the IP_1_ (1% isoflurane and 1.2 μg/ml of propofol) treatment group after hypoxia were negative, while there were significant changes in the IP_2_ (1.4% isoflurane and 0.6 μg/ml of propofol) and IP_3_ (0.5% isoflurane and 1.8 μg/ml of propofol) group. The protective indicator BiP, which can alleviate cell damage by reducing ER stress, was most significantly increased in the IP_1_ group. Therefore, we believe that 1% isoflurane and 1.2 μg/ml of propofol are a better anesthetic choice for neurons already injured by hypoxia.

ER stress is the initial response of cells under stress (Zhao et al., 2016). BiP, the key molecular chaperone in the ER, can help to maintain calcium homeostasis (Ouyang et al., 2011); overexpression or induction of BiP possesses anti-apoptosis potential (Xiao-Hong et al., 2006; Li et al., 2008). The CHOP-mediated pathway is involved in ER stress induced neuronal apoptosis (Oida et al., 2008). In our study, an up-regulated level of BiP suggested the involvement of it in the neuroprotection of 1% isoflurane and 1.2 μg/ml of propofol. After knockdown of BiP-siRNA, expression of CHOP and caspase-12 increased and cell viability decreased distinctly when hypoxic neurons were treated with 1% isoflurane and 1.2 μg/ml of propofol. It is suggested that endogenous BiP plays an important role in the neuroprotection of 1% isoflurane and 1.2 μg/ml of propofol.

Neuronal failure of the proteostasis network may cause protein aggregation that leads to neurodegeneration (Ogen-Shtern et al., 2016; Hetz and Saxena, 2017). The α1 subunit of GABA_A_R, the most prevalent receptor subtype in the brain, is related to cognition (Möhler, 2006; Berry et al., 2008). Neurons expressing α1 GABA_A_R have been found to mediate sedation (Möhler, 2006). In this study, proteostasis of GABA_A_R α1 subunit was used to evaluate the role in the neuroprotection of 1% isoflurane and 1.2 μg/ml of propofol. Results showed that 1% isoflurane and 1.2 μg/ml of propofol enhanced GABA_A_R α1 subunit proteostasis by raising endogenous BiP.

The clinical application value of this study is that it provides a safer anesthetic regimen for elderly patients and patients with reduced cognitive function and neural reserves due to underlying diseases such as chronic cerebral insufficiency, sleep apnea syndrome, and cardiovascular disease. With the improvement of people’s living standards and the progress of medical technology, human society is gradually aging. In clinical practice, anesthesiologists will encounter more patients with fragile brain functions, whose central nervous system is less resistant to damage than normal people. Therefore, we must avoid the cognitive impairment caused by surgery and anesthesia and improve the postoperative quality of life of patients through more careful clinical operation and drug selection.

Taken together, our study confirmed that a combination of 1% isoflurane and 1.2 μg/ml of propofol causes the least damage to hypoxic hippocampal neurons of rats than do other dosages of both two drugs and that endogenous BiP plays an important role in this process.

## Data Availability Statement

The original contributions presented in the study are included in the article/Supplementary Materials, further inquiries can be directed to the corresponding author/s.

## Ethics Statement

The animal study was reviewed and approved by Institutional Animal Care and Use Committee of Tianjin Medical University.

## Author Contributions

HW helped with conception and design; acquisition, analysis, and interpretation of the data; and critical revision of the article and gave the final approval. ZY helped with critical revision of the article and gave the final approval. XB and TL helped with conception and design; acquisition, analysis, and interpretation of the data; and drafting and critical revision of the article and gave the final approval. DG helped with analysis and interpretation of data acquisition, analysis, and interpretation of the data and gave the final approval. CY and XW helped with conception and design and gave the final approval. JW helped with conception and design; analysis and interpretation of the data; and critical revision of the article and gave the final approval. All authors contributed to the article and approved the submitted version.

## Conflict of Interest

The authors declare that the research was conducted in the absence of any commercial or financial relationships that could be construed as a potential conflict of interest.

## Abbreviations

ATF4: Activating Transcription Factor 4
ATF6: Activating Transcription Factor 6
BiP: binding immunoglobulin protein
CHOP: C/EBP homologous protein
ER: endoplasmic reticulum
GABA: γ-aminobutyric acid
GABA_A_R: γ-aminobutyric acid A type receptor
UPR: unfolded protein response
XBP1: X-box binding protein 1.
