# Supplementary material for: 1% Isoflurane and 1.2 μg/ml of Propofol: A Combination of Anesthetics That Causes the Least Damage to Hypoxic Neurons
Source: Front Aging Neurosci. 2020 Nov 16;12:591938. doi: 10.3389/fnagi.2020.591938 (PMC7701289; doi:10.3389/fnagi.2020.591938)
Supplement: Supplementary file 1 [file Table_1.DOCX]

**
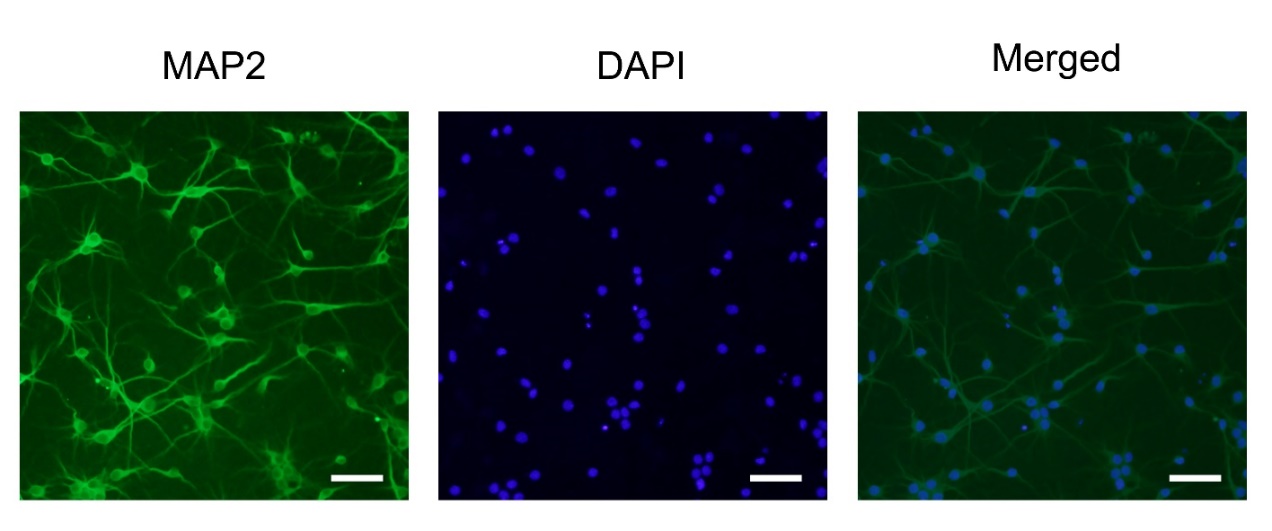
**

**SUPPEMENTAL INFORMATION**

**Figure S1.** **Identification of cultured primary hippocampal neurons.**

Microtubule associated protein 2 (MAP2) and 4,6-diamidino-2-phenylindole (DAPI) were used to identify the purity of cultured primary hippocampal neurons by immunofluorescence. The percentage of cultured neurons is above 95±2.6%. Scale bars = 50 μm
